# Automated and collision-free navigation of multiple micro-objects in obstacle-dense microenvironments using optoelectronic tweezers

**DOI:** 10.1038/s41378-025-00892-9

**Published:** 2025-03-17

**Authors:** Lixiang Zheng, Gong Li, Henan Du, Zonghao Li, Bingrui Xu, Fan Yang, Yanan Mao, Jing Wei, Hainan Xie, Wei Xie, Rongxin Fu, Na Liu, Shuailong Zhang, Lianqing Liu, Wen Jung Li, Yu Sun

**Affiliations:** 1 School of Mechatronics Engineering and Automation, Shanghai University, Shanghai, 200444 China; 2 School of Integrated Circuits and Electronics, Beijing Institute of Technology, Beijing, 100081 China; 3 Zhengzhou Research Institute, Beijing Institute of Technology, Henan, 450000 China; 4 Beijing Advanced Innovation Center for Intelligent Robots and Systems, School of Mechatronical Engineering, Beijing Institute of Technology, Beijing, 100081 China; 5 Optoseeker Biotechnology (Shenzhen) Co., Ltd, Shenzhen, 518055 China; 6 School of Medical Technology, Beijing Institute of Technology, Beijing, 100081 China; 7 Chongqing Institute of Microelectronics and Microsystems, Beijing Institute of Technology, Chongqing, 400000 China; 8 State Key Laboratory of Robotics, Shenyang Institute of Automation, Chinese Academy of Sciences, Shenyang, 110016 China; 9 Department of Mechanical Engineering, City University of Hong Kong, Hong Kong, China; 10 Department of Mechanical and Industrial Engineering, University of Toronto, Toronto, ON M5S 3G8 Canada

**Keywords:** Micro-optics, Electrical and electronic engineering

## Abstract

Automated parallel manipulation of multiple micro-objects with optoelectronic tweezers (OET) has brought significant research interests recently. However, the parallel manipulation of multiple objects in complex obstacle-dense microenvironment using OET technology based on negative dielectrophoresis (nDEP) remain a big technical challenge. In this work, we proposed an adaptive light pattern design strategy to achieve automated parallel OET manipulation of multiple micro-objects and navigate them through obstacles to target positions with high precision and no collision. We first developed a multi-micro-object parallel manipulation OET system, capable of simultaneous image processing and microparticles path planning. To overcome microparticle collisions caused by overlapping light patterns, we employed a novel adaptive light pattern design that can dynamically adjust the layout of overlapping light patterns according to surrounding environment, ensuring enough space for each microparticle and preventing unintended escapes from the OET trap. The efficacy of this approach has been verified through systematic simulations and experiments. Utilizing this strategy, multiple polystyrene microparticles were autonomously navigated through obstacles and microchannels to their intended destinations, demonstrating the strategy’s effectiveness and potential for automated parallel micromanipulation of multiple microparticles in complex and confined microenvironments.

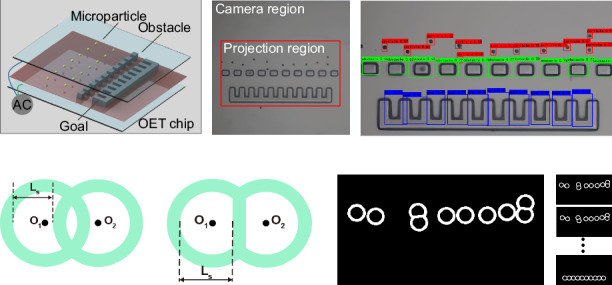

## Introduction

Optoelectronic tweezers (OET) represent a reliable form of optical micromanipulation that utilizes the optically induced dielectrophoresis (DEP) phenomenon to manipulate nanoscale and microscale objects^1–3^. Compared to widely used optical tweezers (OT)^4,5^ the OET technology operates with a significantly lower optical power density ( < 1 W/cm²)^6^, thereby minimizing photothermal effects^7^. Additionally, OET provides stronger manipulation forces and higher throughput, enabling it to manipulate a variety of nano-/micro-objects, including nanoparticles^8,9^, nanowires^10,11^, microparticles^12–15^, cells^16–20^, microrobots^21–24^, microtissues^25^ and photonic components^26,27^.

Driven by the need for high-throughput, single-cell-level parallel manipulation in biomedical applications, the automation and parallelization of multiple micro-object manipulation using OET have recently gained significant attention^28–35^. Despite advancements in automation, the coordinated use of negative DEP force to simultaneously manipulate multiple particles in a confined and cluttered space remains a significant challenge. While manipulation based on negative DEP force can provide a separate chamber for each particle to ensure single-cell-level precision control, the size of the light pattern used to manipulate particles is typically five to ten times larger than the particles themselves. This discrepancy frequently leads to overlapping light patterns, causing micro-objects to collide and inadvertently escape their traps. A conventional mitigation technique involves using the outer radius of the light pattern as the collision radius for path planning algorithms. However, this method increases computational load and fails to secure reliable navigation paths in obstacle-dense environments, highlighting a significant gap in achieving reliable automated manipulation.

**Fig. 1:**
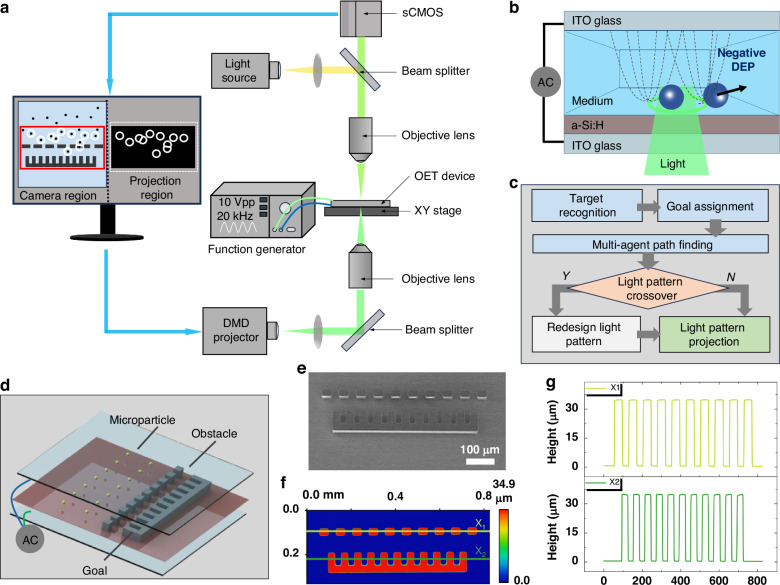
Experimental setup and device structure. **a** Schematic experimental setup. **b** Schematic of the OET device, showing manipulation of microparticles via negative DEP forces. **c** Flowchart of the OET automatic manipulation system. **d** 3D schematic the OET device with microstructures. **e** SEM image of the fabricated microstructures. **f** 3D optical profile of the fabricated microstructures. **g** Horizontal height distribution of microstructures

This study presents an adaptive light pattern design strategy for high-efficiency, multi-objective path planning and autonomous manipulation in complex, obstacle-rich environments, as illustrated in Fig. 1. The system setup, shown in Fig. 1a, includes a computer that acquires images from a camera and projects light pattern sequences to achieve parallel, automated control of multiple objects. Under the specified conditions, the manipulated object is subjected to negative DEP forces, either confined within or repelled by the light pattern (Fig. 1b). The schematic control diagram of the OET manipulation system (Fig. 1c) comprises four main components: target recognition, goal assignment, multi-agent pathfinding, and an adaptive light pattern design unit. In a key experiment, ten 10-µm polystyrene (PS) microparticles were successfully transported to pass microchannels to designated microchambers within an obstacle-dense environment (Fig. 1d–g), reaching their target positions without loss, demonstrating the strategy’s effectiveness and stability. This adaptive light pattern design approach has considerable potential for advancing OET-based automated micromanipulation in complex microenvironments, representing a pivotal innovation in the field.

## Results

### Detailed strategy for multi-objects manipulation

Figure 1c presents the schematic control diagram of the OET manipulation system, which consists of four main components: the detection unit, the goal assignment unit, the path finding unit, and the adaptive light pattern design unit.

1. Targets and obstacle detection: As the projected light pattern is smaller than the camera’s field of view in our OET system, it is necessary to crop the captured images to align with the maximum area of the projected light pattern. This cropping is achieved through classical image processing techniques using the OpenCV library. Subsequently, the microparticles, obstacles, and microchambers within the cropped images are identified and labeled using the YOLOv7 object detection algorithm^36,37^, an advanced deep learning model widely used for object detection tasks.
2. Goal assignment: For multi-microparticle manipulation, determining the optimal path between the microparticles and the microchambers is crucial. In this work, the Hungarian algorithm^38,39^ was used to assign each microparticle to its corresponding microchamber based on the global minimum cost, calculated using the Euclidean distance between the starting position and the microchamber’s position (final position). In this case, the goal is to find a permutation σ that minimizes the minimum cost of the following function:
  1 $$\sigma ={argmin}\mathop{\sum }\limits_{i=1}^{n}{c}_{i,\sigma (i)}$$
  where n is the size of the cost matrix, ${c}_{i,\sigma (i)}$ is an element in the cost matrix *C*, representing the cost in row i and column $\sigma (i)$. The cost matrix *C* can be expressed as:
  2 $$C=\left[\begin{array}{cccc}{D}_{1-1} & {D}_{1-2} & \cdots & {D}_{1-n}\\ {D}_{2-1} & {D}_{2-2} & \cdots & {D}_{2-n}\\ \vdots & \vdots & \ddots & \vdots \\ {D}_{n-1} & {D}_{n-2} & \cdots & {D}_{n-n}\end{array}\right]$$
  where ${D}_{i-j}$ is the Euler distance between the starting position of the i-th microparticle to the position of j-th micro-chamber.
3. Multi-agent pathfinding: The classical Conflict-Based Search (CBS) algorithm^40^ is employed. CBS operates on two levels: the low level, where each agent independently computes its path using shortest-path algorithms like A*, and the high level, which examines these paths for conflicts where agents occupy the same location at the same time step. When a conflict is detected, constraints are applied to the conflicting agents, prompting a re-execution of the low-level search to generate conflict-free paths. This iterative process continues until all paths are conflict-free. Increasing the safety distance *d*_*s*_ heightens the likelihood of conflicts, leading to more frequent cycles of conflict detection, constraint application, and path re-computation. Consequently, computational complexity rises, potentially obstructing feasible path calculation. This issue is further intensified in obstacle-rich environments, where search space limitations necessitate a smaller *d*_*s*_ to maintain efficient computation of feasible, conflict-free paths. Detailed mathematical principles underlying multi-agent pathfinding are presented in the Methods section.
4. Adaptive light pattern design: The adaptive pattern design strategy initially checks if the center-to-center distance of any two light patterns is less than the critical crossing distance. In the case of two light patterns crossing and overlapping, the light pattern is redesigned to ensure sufficient space for microparticle manipulation. In the case that there is no overlap between the light patterns, the original ring light pattern is retained. Upon applying the adaptive light pattern design strategy, the preprogrammed light pattern sequences are projected on the OET chip to ensure stable trap and manipulation of microparticles.

### The influence of the light pattern’s inner diameter on manipulation performance

**Fig. 2:**
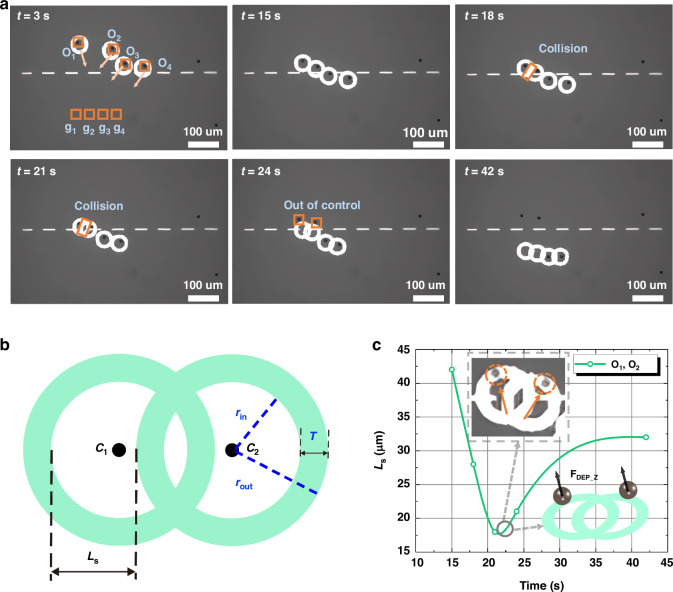
Experiments of transporting microparticles to specified locations, along with an analysis of failure causes. **a** Four microparticles were targeted for transport; however, the process failed for the *O*_*1*_ and *O*_*2*_ microparticle due to interference from overlapping light patterns (supplementary Movie S1). **b** A schematic depicts the intersection and overlap of light patterns with two green rings representing the light patterns. The inner and outer radii of the rings are denoted as *r*_*in*_ and *r*_*out*_, respectively, with *T* representing the thickness of the rings. The distance between *C*_*1*_ and *C*_*2*_. is labeled as *d*. *L*_*s*_, which represents the available space used for microparticle manipulation, varies depending on the distance between the centers of the two ring patterns. *L*_*s*_ indicates the degree of overlap. **c** The temporal variation of *L*_*s*_ for the *O*_*1*_ and *O*_*2*_ microparticle is presented. The inset shows the moment at 21 seconds when the two microparticles escaped the confinement of the ring light patterns, along with a schematic diagram illustrating the upward forces acting on the particles at that time

Figure 2a demonstrates the automated manipulation of four 10 μm PS microparticles without the adaptive light pattern design strategy (as further detailed in Movie S1). The images timestamp the process, while ‘*O*_*i*_’ and ‘*g*_*i*_’ mark the center and final positions of each microparticle, respectively. The white rectangular light patterns represent virtual obstacles in the center of the image. From t = 3 s to t = 15 s, the circular ring light patterns did not overlap or intersect, allowing for controlled manipulation of the microparticles. At t = 18 s, the light patterns for the *O*_*1*_ and *O*_*2*_ microparticles neared each other, causing a partial intersection and reduction of the dark central areas of the ring light patterns. However, control over the microparticles was maintained. By t = 21 s, further intersections and overlaps compromised control due to significant reduction in the dark central areas of the ring light patterns. At t = 24 s, the extensive overlap and collision diminished the trapping forces on the *O*_*1*_ and *O*_*2*_ microparticles, leading to their escape from the trap. By t = 42 s, only the *O*_*3*_ and *O*_*4*_ microparticles were successfully transported to their designated microchambers (target positions).

Analyzing the loss of control of microparticles due to the intersection of light patterns is important for enhancing the capability of OET when it is used for parallel manipulation of multiple microparticles. Figure 2b illustrates two intersecting ring light patterns. The two green rings and associated parameters provide details of the used light pattern. *C*_*1*_ and *C*_*2*_ represent the centers of light patterns 1 and 2, respectively. The ring light patterns are characterized by their inner radius *r*_*in*_ and outer radius *r*_*out*_. The size of *r*_*in*_ defines the central manipulation area, while *r*_*out*_ affects the ring’s thickness *T* (*T = r*_*out*_ *– r*_*in*_), which influences the manipulation force^41^. *L*_*s*_ is used to indirectly represent the size of the light pattern’s internal chamber. When the distance *d* between *C*_*1*_ and *C*_*2*_ exceeds the critical conflict distance *d*_*c*_ (where *d*_*c*_ *= r*_*in*_ *+ r*_*out*_) of the two light patterns, no conflict/collision occurs between the light patterns, and *L*_*s*_ *= 2r*_*in*_. As the center-to-center distance *d* decreases, *L*_*s*_ also decreases. When *d* is less than *d*_*c*_, *L*_*s*_ can be described as:

3 $${L}_{s}=2{r}_{{in}}-\left({d}_{c}-d\right)=d-T$$

Based on Eq. (3), T is a predefined constant value, *L*_*s*_ decreases as the center-to-center distance *d* decreases. A safe distance *d*_*s*_ is defined as the minimum allowable distance between two light patterns, where *L*_*s*_ *= d*_*s*_ *– T*.

Figure 2c illustrates the temporal change of *L*_*s*_ as the *O*_*1*_ and *O*_*2*_ microparticle navigate through a microchannel between obstacles. Over time, *L*_*s*_ keeps on decreasing, reaching a minimum at 21 s. At this point, the inhomogeneous electric field near the virtual electrode generated by the light pattern is unable to produce a stable horizontal negative DEP force due to the reduced manipulation area. Consequently, the particles fail to follow the movement of the light pattern as expected. The experimental results in Fig. 2c, along with the force schematic, show that when the particles are located on top of the optical pattern ring, they experience a vertical DEP force along the Z-axis. This force propels the particles upward, causing them to lose focus and escape from the light pattern with a hop^42^. These findings demonstrate that a safe distance *d*_*s*_ of 10 μm is inadequate for ensuring effective manipulation of multiple microparticles in environments filled with obstacles where physical collisions between microparticles are unavoidable.

**Fig. 3:**
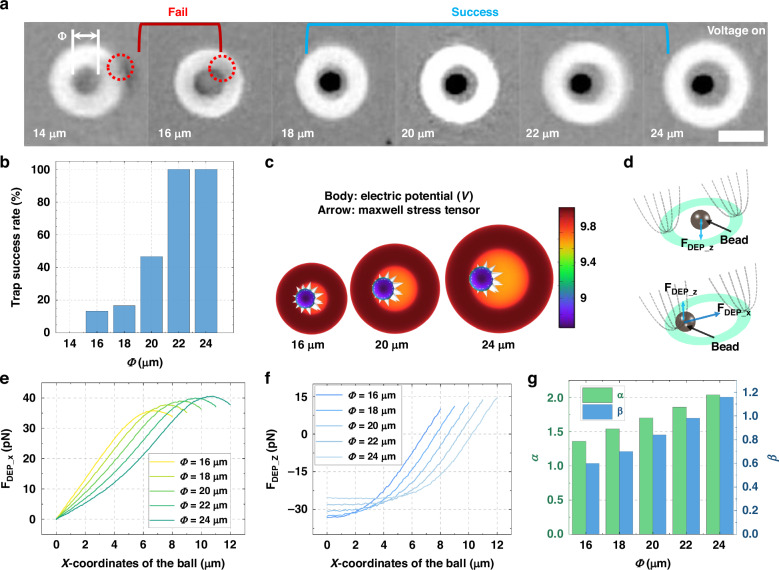
The effect of inner diameter on the manipulation performance of light patterns. **a**. Microscopic images of 10 μm PS microparticles trapped by ring light patterns with the same thickness T but varying inner diameters $\Phi$ (i.e., 16 μm), scale bar 25 μm. Light patterns with inner diameters of 16 μm and 18 μm failed to trap the particles, whereas other patterns successfully did so. **b** Success rate of trapping versus light pattern’s inner diameters $\Phi$. **c** Distribution of electric potential and Maxwell stress tensor for a single microparticle at the edges of light patterns with different inner diameters. **d** Schematic diagram illustrating the forces acting on a microparticle positioned at both the edge and center of the ring light pattern. **e** Horizontal and **f** vertical DEP force distributions on a microparticle located at various positions within light patterns of different inner diameters. The center of the light pattern is denoted as position 0. **g** Relationship between α, β (indicating the stable controllable range of the internal chamber of the light pattern) and the inner diameters of the light patterns

To find the appropriate light pattern size for stable manipulation of PS particle, light patterns with various inner diameters were used to trap 10 μm microparticles to study the effect of the inner diameter of the light pattern on the manipulation performance, as shown in Fig. 3a. The corresponding trapping success rates are presented in Fig. 3b. For light patterns with inner diameters (Φ) of 16 μm and 18 μm, the success rate was below 20%, which is insufficient for practical use. In contrast, a light pattern with a diameter of 20 μm achieved a success rate of approximately 46.67%, and for diameters of 22 μm and 24 μm, the success rate reached 100%. (The raw experimental data corresponding to Fig. 3b can be found in Table S1 in the supplementary information).

To investigate the physical mechanisms underlying the observed experimental results, simulations were performed using the AC/DC module in COMSOL Multiphysics (more details in Method Section). The behavior of 10 μm PS microparticles under different ring light patterns with inner radii of 16, 18, 20, 22, and 24 μm and a thickness (*T*) of 10 μm was analyzed. Figure 3c shows the distribution of the electric potential and Maxwell stress tensor for a single PS microparticle at the edge of light patterns with varying inner diameters. Based on the distribution of the Maxwell stress tensor^43,44^, it can be observed that the region of the light pattern near the microparticle exerts a repulsive force on it, with the net force directed towards the interior of the light pattern. However, for optical patterns with smaller inner diameters, the light pattern on the opposite side of the particle can have a non-negligible effect on the particle. Figure 3d presents a force analysis of the microparticle at different positions within the light pattern. As shown, at the center of the pattern, the repulsive forces from the virtual electrodes cancel each other out in the horizontal direction, while in the Z-axis direction, the repulsive forces are additive, resulting in a downward dielectrophoretic (DEP) force. At the edge of the light pattern, the repulsive force exerted by the virtual electrode on the same side in the horizontal direction is significantly greater than that on the opposite side. Similarly, in the vertical direction, the upward repulsive force from the virtual electrode on the same side is much stronger than the downward force on the opposite side. As a result, the microparticle experiences a repulsive force pushing toward the center in the horizontal direction and an upward lifting force in the vertical direction.

Figure 3e, f show the horizontal and vertical DEP forces exerted on the microparticle at various positions within circular ring light patterns with different Φ from the center to the edge. A light pattern with a smaller Φ makes it easier for microparticles to be distributed at the edge of the light pattern during the capture process, resulting in a stronger upward lifting force and easy manipulation failure. Additionally, the smaller Φ results in a larger gradient in the repulsive force exerted on the microparticles, making the manipulation process unstable in the horizontal plane. To quantify the influence of different inner diameters on particle manipulation, we define two parameters: *α* and *β*. *α* is the ratio of the area where a microparticle experiences a downward vertical force to the size of the sphere (higher *α* values indicate greater stability in both the capture and manipulation processes.). *β* is the ratio of the area within the ring where the horizontal force on the sphere is less than or equal to 20 pN to the size of the sphere (higher *β* values correspond to a larger buffer zone near the center of the ring, where the horizontal DEP force is minimal, promoting operational stability). Figure 3g illustrates that both *α* and *β* values increase as the inner diameter increases. This observation is consistent with the experimental results, indicating that a certain internal chamber size within the light pattern is essential for effective and stable manipulation. Based on above finding, light pattern with 22 μm was selected for the PS microparticle manipulation.

### Adaptive light pattern design strategy

**Fig. 4:**
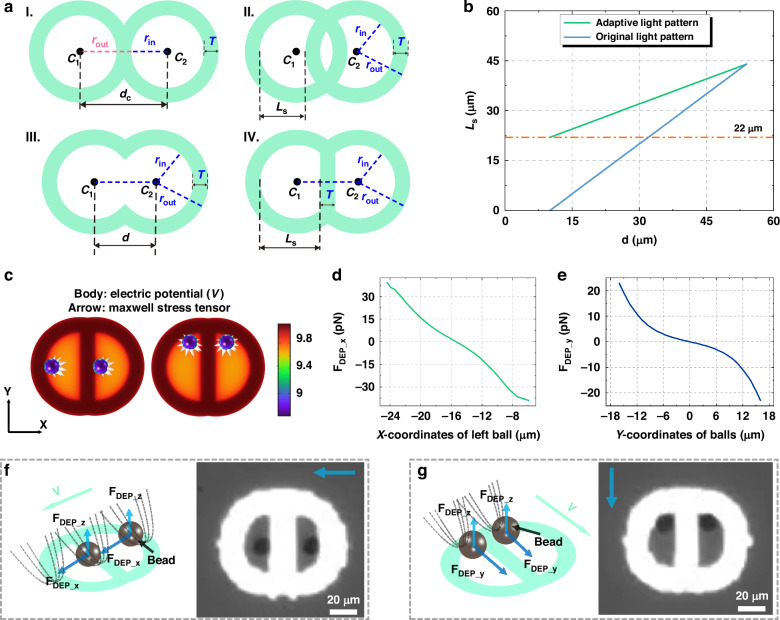
Adaptive light pattern strategy for OET manipulation and its analysis. **a** The adaptive light pattern design process. **b** Curves representing *L*_*s*_ as a function of the center-to-center distance *d*, shown both before and after the implementation of the adaptive light pattern design strategy. With the adaptive approach, the minimum *L*_*s*_ is maintained at 22 µm, ensuring robust microparticle manipulation. In contrast, the non-adaptive pattern results in a minimum *L*_*s*_ of 0 µm, leading to ineffective control and potential microparticle escape. **c** Electric potential and Maxwell stress tensor distributions for two microparticles positioned at different locations within the adaptive light pattern. **d** DEP force distribution along the X-axis and **e** Y-axis for a microparticle located at various positions in the left half of the adaptive light pattern. **f** Force analysis and corresponding microscope images of two particles moving leftward and **g** downward under the adaptive light pattern strategy (supplementary Movie S2)

To resolve the manipulation failures caused by the overlapping of two ring light patterns, our initial strategy involved using the outer radius of the light patterns as the collision radius in the path planning algorithm. However, the radius exceeded the width of the channel between obstacles, complicating the route planning for the microparticles. To overcome this, we developed an adaptive light pattern design strategy specifically designed to address the manipulation failures due to cross-interference of light patterns. Figure 4a I displays the two ring light patterns at the critical intersection distance *d*_*c*_, and Fig. 4a II shows the intersection of these patterns, with *L*_*s*_ indicating the space available for microparticle manipulation. In Fig. 4a III and 4a IV, the process of the adaptive light pattern design is presented: initially, the intersection area is cleared to mitigate its impact on microparticle manipulation. We then introduce a 10-μm-thick rectangular boundary at the center of each ring light pattern to create separate manipulation spaces for each light pattern. For this adaptive light pattern design strategy, *L*_*s*_ can be expressed as:

4 $${L}_{s}={r}_{{in}}+0.5\left(d-T\right)$$

where *r*_*in*_ represents the inner radii of the circular ring light pattern, *d* denotes the distance between the centers of the two circular ring light patterns, and *T* stands for the thickness of the circular ring light pattern. Figure 4b presents the *L*_*s*_ curves for both implementations of the adaptive light pattern design strategy—utilized and not utilized. The data clearly show that employing the adaptive strategy yields consistently higher *L*_*s*_ values for any given center-to-center distance *d* compared to the non-adaptive approach. Notably, the adaptive strategy ensures that *L*_*s*_ maintains a minimum value of 22 μm when the distance *d* reaches its minimum value of 10 μm, thereby guaranteeing effective and reliable microparticle manipulation with no collision. In contrast, without this strategy, *L*_*s*_ can drop to 0 μm, resulting in insufficient manipulation space and subsequent escape of the microparticle from the OET trap, as demonstrated in experimental observations.

To assess the effectiveness of the adaptive light pattern design strategy, comprehensive simulations, force analyses, and experimental validations were systematically conducted. Both experiments and simulations involved two significantly overlapping light patterns, with a center-to-center distance *d* of 10 μm and an effective manipulation space *L*_*s*_ of 22 μm. These parameters represent the minimal operational conditions for the adaptive light pattern design. Figure 4c illustrates the electric potential distribution around two microparticles positioned at the left and top of the light pattern. Figure 4d, e detail the distribution of DEP forces along the X and Y axes, respectively, as the microparticle occupies various positions within the light pattern. At an X coordinate of -15 μm and a Y coordinate of 0 μm, the microparticle experiences zero net force, indicating stable positioning within the OET trap under a stationary light pattern. It is evident that due to the adaptive light pattern design strategy, which modifies the shape and arrangement of the light pattern, the DEP force along the X-axis near the edge is greater than that along the Y-axis, though this does not affect the manipulation outcome. Figure 4f, g show the DEP force analysis and corresponding microscope images of microparticles moving in the X and Y directions at a speed of 10 μm/s, with further details available in Movie S2. As anticipated, the light patterns exert forces that guide the microparticle towards the center of the manipulation area. These experimental and simulation results confirm the efficacy of the adaptive light pattern design for microparticle manipulation. This strategy not only reduces the computational complexity but also enhances the success rate of parallel OET manipulation, effectively preventing target loss caused by overlapping light patterns.

### The performance of adaptive light pattern design strategy

**Fig. 5:**
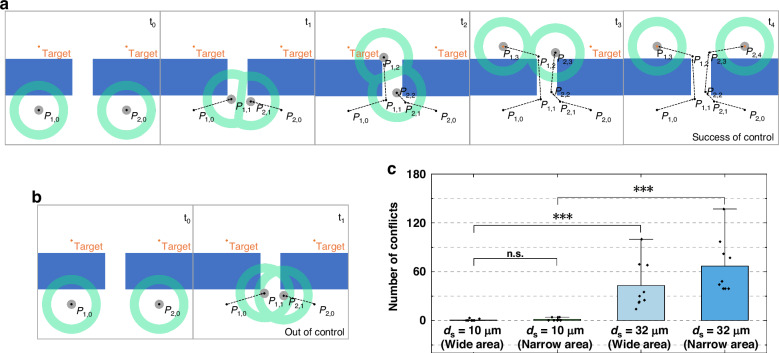
Simulation of path planning using the adaptive light pattern design strategy. **a** The path planning process in a constrained space compares the performance of the adaptive light pattern strategy with **b** the original, non-adaptive approach. The adaptive strategy effectively reduces the distance between microparticles while maintaining control, demonstrating its efficacy in confined spaces. **c** The number of path conflicts between neighboring microparticles is compared using adaptive and original light patterns across different application scenarios

Figure 5a, b present the schematics of path planning using the Conflict-Based Search (CBS) algorithm for two microparticles passing through a narrow channel (details of the CBS algorithm and how it is implemented can be found in the Method section). This simulation highlights the efficacy of the adaptive light pattern strategy, which dynamically facilitates the fusion of neighboring light patterns to increase *L*_*s*_ and ensure successful manipulation. At time *t*_*1*_, when the microparticles are closest, the adaptive strategy’s larger *L*_*s*_ ensures that the particles continue to follow their designated paths (Fig. 5a). Conversely, the original light pattern strategy struggles, as a reduction in *L*_*s*_ leads to manipulation failure and microparticles out of control (Fig. 5b). This necessitates the use of larger safe distances between paths in the traditional strategy, thereby escalating computational complexity. The adaptive light pattern design allows for a safety distance *d*_*s*_ of 10 μm in the CBS multi-objective path planning algorithm, ensuring that *L*_*s*_ >= 22 μm. In contrast, the original light pattern design requires a minimum safety distance *d*_*s*_ of at least 32 μm to guarantee *L*_*s*_ >= 22 μm. A larger safety distances *d*_*s*_ significantly increases the number of conflicts during the path planning process. As depicted in Fig. 5c, the adaptive strategy significantly enhances algorithmic efficiency by reducing number of path conflicts between two neighboring microparticles, a benefit that becomes particularly evident in narrow and crowded areas. Excessive path conflicts in the traditional approach can prolong computational time and potentially exceed the maximum cycle threshold set by the algorithm, leading to non-convergent results. Thus, the adaptive light patterning strategy offers substantial advantages for parallel manipulation of multiple microparticles in environments dense with obstacles and narrow channels, optimizing both computational efficiency and manipulation success rate.

**Fig. 6:**
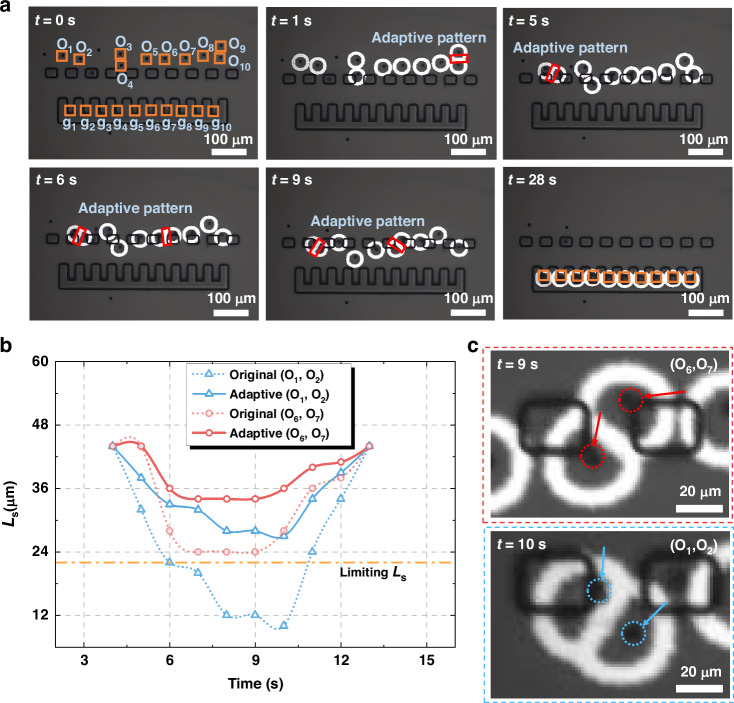
Application of adaptive light patterns for automatic transportation of 10 microparticles in an obstacle-dense environment. **a** Microscope images showing the transport of 10 PS microparticles through microchannels between obstacles to the microchambers (supplementary Movie S3). **b** The variation of L_s_ over time for the original and adaptive light patterns used to transport *O*_*1*_ and *O*_*2*_, as well as *O*_*6*_ and *O*_*7*_. **c** Microscope images depicting the closest proximity between the *O*_*1*_ and *O*_*2*_ at 11 s, and between *O*_*6*_ and *O*_*7*_ at 9 s, demonstrating manipulation using adaptive light patterns

Figure 6a displays the experimental results of autonomous navigation of ten 10 µm diameter PS microparticles to reach designated microchambers at a speed of 10 µm/s using an adaptive light pattern, as detailed in Movie S3. At t = 0 s, the center (‘*O*_*i*_’) and final (‘*g*_*i*_’) positions of each microparticle are marked by orange rectangles, with rectangular microstructures representing physical obstacles. By t = 1 s, all ten microparticles are successfully trapped within ten circular ring light patterns, despite a slight overlap between the patterns of the *O*_*9*_ and *O*_*10*_ microparticles. From t = 5 s to t = 9 s, overlaps between the light patterns for the *O*_*1*_ and *O*_*2*_, as well as the *O*_*6*_ and *O*_*7*_ microparticles, intensify significantly. The adaptive light pattern design strategy dynamically adjusts the layouts to mitigate the effects of overlap and maintain adequate manipulation space. By t = 28 s, all microparticles successfully reach their targeted positions within the microchambers. Figure 6b compares the changes in *L*_*s*_ from t = 4 s to t = 13 s between the original and adaptive light patterns for the *O*_*1*_, *O*_*2*_, *O*_*6*_, and *O*_*7*_ microparticles, demonstrating that *L*_*s*_ is consistently larger with the adaptive patterns, providing stable manipulation. Figure 6c presents experimental images capturing the minimum distance between neighboring microparticles (*O*_*1*_ and *O*_*2*_, *O*_*6*_ and *O*_*7*_) during manipulation by adaptive light patterns. These images demonstrate that the microparticles remain controlled and navigate successfully through narrow channels between obstacles. These results underscore the efficacy of the adaptive light pattern strategy in enabling precise, reliable, and automated manipulation of multiple microparticles in complex microenvironments filled with obstacles and microchannels. It is important to note that, based on user requirements, an open target selection module can be provided to customize the target positions for various objects (see supplementary Movie S4). This feature has potential applications in fields such as biomedicine, microbiology, and related areas.

## Discussion and conclusion

In this study, we developed an automated OET manipulation system that integrates image recognition and path planning to identify and localize microparticles, obstacles, and microchambers, assigning each microparticle a specific path and destination. A series of circular ring light patterns, generated by the path planning unit, is projected onto the OET device to autonomously guide microparticle movement. To prevent manipulation failures due to overlapping light patterns in obstacle-dense microenvironments, we examined the correlation between light pattern inner diameter and controllability, identifying the parameter *L*_*s*_ as the minimum inner diameter required for stable manipulation. Based on this insight, we developed an adaptive light pattern design strategy that dynamically monitors the center-to-center distances between light patterns, detecting intersections and adjusting patterns as needed to maintain adequate manipulation space. Our results demonstrate that this adaptive strategy significantly optimizes spatial utilization, enhancing both manipulation efficiency and success rate. Experiments with ten PS microparticles in a microenvironment filled with obstacles and microchannels confirmed the approach’s efficacy. All microparticles were successfully transported to designated positions along predefined paths.

For the parallel manipulation of multiple objects, the proposed scheme effectively addresses common conflict scenarios, particularly those where two objects direct interfere with each other—these are among the most frequent and significant types of conflicts. We also considered that conflict scenarios involving more than two objects could occur, though with a much lower probability due to the use of the above algorithm. To address the conflicts involving more than two objects, we did some preliminary work based on simulation as presented in the Supplementary Information. Briefly, these conflicts can be categorized into two types: individual two-object conflicts and multiple two-by-two object conflicts. By addressing both cases, the adaptive light pattern design strategy can be generalized to ensure stable manipulation. The Supplementary Note 1 details the adaptive light patterning strategy for each case and discusses its advantages in improving computational efficiency (by minimizing path conflicts through reduced safety distances) while ensuring manipulation stability with sufficient *L*_*s*_ values. In most scenarios, the pathfinding algorithm minimizes multi-object encounters, typically limiting conflicts to only two objects. As the number of manipulated objects increases and the available operational space decreases, the likelihood of multi-object conflicts rises, making the proposed method in the Supplementary· Information potentially useful. However, in practical applications, experimental conditions are typically optimized to keep the number of objects manageable and the operational space sufficiently uncrowded, thereby reducing computational and experimental complexity. Consequently, conflict scenarios involving direct interference between two objects (the case reported in the main text) remain the primary cases to consider and address.

It should be noted that to address the issue of collisions between light patterns during multi-particle parallel manipulation, the adaptive light pattern design strategy presented in this paper leverages the flexible characteristics of light manipulation. This strategy optimizes the design of light patterns in the light pattern design unit, enabling automatic reconstruction of the light patterns upon collision, thereby ensuring sufficient manipulation space. In contrast, path planning algorithms typically resolve light pattern collisions by increasing the safe distance *d*_*s*_ of each target or adopting more complex path calculation strategies, which can result in higher computational costs or even unconverging solutions. The adaptive light pattern design strategy effectively resolves these challenges by shifting the problem from path planning to the light pattern design module. This method is simple, efficient, and can be combined with various target assignment and path planning algorithms to maximize the potential and advantages of OET in multi-target parallel manipulation.

Overall, this study not only showcases the adaptability of OET technology but also highlights its potential for automated parallel manipulation of microscale objects in complex environments, offering a practical solution where conventional methods fail to work. This approach significantly reduces computational complexity and minimizes target loss due to cross-interference between light patterns, thereby enhancing the universality OET and fostering its broader and more effective applications in micromanipulation. However, there is still room for improvement in the experimental design and methodology presented in this paper. Future work could focus on increasing the number of particles, introducing obstacles of various shapes, altering the spatial arrangement of obstacles, and customizing the manipulation of different particle types. These improvements would better align the study with the complex application requirements of integrating with microfluidic technologies in practical scenarios.

## Methods

### Experimental setup and device structure

Figure 1a illustrates the configuration of the OET system, which features a digital micromirror device (DMD) projector (Mightex Polygon 400 with a 625 nm 1100 mW LED source) for projecting dynamic light patterns onto the OET chip via a 50X objective lens. The optical power density of the light pattern projected through the objective lens was measured using a Thorlabs PMl6-130 power meter, with an optical power density of 0.63 W/cm^2^ (no significant photoinduced heating was observed under these conditions). The system uses a function generator (Agilent 33522 A) to power the OET chip, which is positioned on a three-dimensional motorized stage (Tango Desktop). Above the chip, a sCMOS camera (Teledyne Iris OPTIMOS-F-M-16-C) captures real-time images through a 10X objective lens. This setup was used for the majority of the OET experiments except for the one shown in Movie S4. The computer configuration used in this study includes 8 GB of RAM, an Intel Core i5-6300 CPU, and an NVIDIA Quadro P620 GPU. Image preprocessing and adaptive light pattern design are implemented using the C + + language combined with OpenCV and Eigen library. The yolov7 target recognition algorithm, target allocation algorithm, and CBS path planning algorithm are sourced from github open-source and deployed on the local computer.

As illustrated in Fig. 1b, d, the OET chip consists of top and bottom glass substrates, both coated with indium tin oxide (ITO). The bottom substrate is further coated with a 1 μm-thick layer of hydrogenated amorphous silicon (a-Si:H). The two substrates are assembled using 150 μm thick double-sided tape, forming a microchamber for micromanipulation. The inner picture in Fig. 1e, f and Fig. 1g illustrate the 3D microstructures fabricated on the bottom substrate of the OET chip, which include obstacles and micro-chambers. The spacing between adjacent obstacles and the internal dimensions of each micro-chamber are 30 µm. These microstructures at 30 µm in height, exceeding the size of the microparticles, effectively preventing them from moving directly through the obstacles into the microchambers.

### Microfabrication

The obstacles and microchambers on the OET chip were created using SU-8 2015 photoresist (MicroChem). Initially, 4 mL of SU-8 2015 was spin-coated at 1300 rpm to achieve approximately 30 μm-thick microstructures, and this was done for 30 s atop the OET bottom plate. This step was followed by a soft bake at 65 °C for 3 min and then at 95 °C for 8 min. A mask aligner (URE-2000/35) then illuminated the substrates for 10 s at an exposure energy of 9 mJ/cm² using a photomask to selectively photo-crosslink the SU-8. After exposure, the substrates were baked again at 65 °C for 2 min and 95 °C for 7 min, then developed in SU-8 developer for 8 min. Following development, the microstructures on the OET bottom plate were air-dried using pressurized nitrogen. For experiments, spherical polystyrene (PS) microparticles 10 µm in diameter (Aladdin) were suspended in deionized water with 0.05% v/v Tween 20 (T8820 Solarbio). Typically, 10 µL of this suspension was pipetted into the chamber of the OET device for each experiment. The OET device is intended for single use to avoid cross-sample contamination

### Manipulation mechanism and numerical simulations

The functionality of the OET device relies on the photoconductive properties of the a-Si:H layer. In the absence of light, the a-Si:H layer exhibits high impedance, causing most of the voltage to drop across it. However, when illuminated, the impedance of the a-Si:H layer decreases significantly, shifting the voltage drop to the liquid medium above. As a result, projecting light patterns with illuminated and dark regions onto the a-Si:H layer creates a non-uniform electric field within the liquid medium (Fig. 1c). This field interacts with microparticles suspended in the medium, generating DEP forces—either repulsive (negative DEP) or attractive (positive DEP). It is important to note that the conductivity of the solution should not exceed that of a-Si:H, as this would hinder the application of the electric field to the solution layer, making it difficult to generate sufficient manipulation force, according to impedance matching theory^45^. In this work, a negative DEP force is applied to manipulate PS particles. The use of light patterns instead of focused light beams offers a significant advantage for OET technology, enabling DMD projectors to generate hundreds or even thousands of optical traps with low power intensity. This capability allows for high-throughput, simultaneous manipulation of multiple micro-objects. While this functionality is essential, it also requires the development of more sophisticated parallel control algorithms to manage these operations effectively.

A simulation model has been built to analyze the DEP force experienced by the PS microparticles in OET device. The DEP force applied to the PS microparticle could be obtained by integrating the Maxwell stress tensor (MST) over the surface of the PS microparticle, which could be expressed as^43,44^:

5 $${F}_{{DEP}}=\oiint T\,\cdot\, \mathop{n}\limits^{ \rightharpoonup }{dS}$$

where *T* represents the MST on the PS microparticle’s surface, $\mathop{n}\limits^{ \rightharpoonup }$ represents the unit vector normal to the PS microparticle’s surface, ${dS}$ represents the area element of the PS microparticle’s surface.

Simulation of the OET device was built as a 3D Alternating Current (AC) module using COMSOL Multiphysics. Here, the photoconductive(a-Si:H) layer and the liquid media were set as 1 μm and 150 μm in the model. The coordinate origin was set at the center of the light pattern. The PS microparticle of 10 μm in diameter was set 1 μm above the a-Si:H. Regions of high conductivity are consistent with experiments. The conductivity and permittivity of materials can be found in Table S2 in the Supplementary· Information. In this AC module, the electric potential of the bottom and top ITO electrode was set as 10 V and 0 V, respectively. The AC frequency was set to 20 kHz, consistent with the experiment. Based on this, the electric field distribution and the MST and charge distribution on the PS microparticle’s surface can be simulated by solving the continuity equation of the AC module, which could be used to calculate the DEP force exerted on the PS microparticle.

The microparticles used in this experiment feature a hollow spherical shell structure, resulting in a lower conductivity compared to solid PS microparticles, due to the influence of the air medium inside^46,47^. It is important to note that when the microparticle conductivity is lower than that of the solution, they experience negative DEP forces^48,49^. As shown in Supplementary Note 2 and Fig. S3, variations in the conductivity magnitude of the microparticles within the range of negative DEP do not affect the numerical simulation results significantly.

### Multi-agent pathfinding

Finding routes from multiple microparticles to the positions of micro-chambers can be simplified as a multi-agent pathfinding (MAPF) issue^50^. The input to the multi-agent pathfinding issue (MAPF) is:

- An undirected graph $G=(V,E)$, the vertices $v\in V$ of the graph are possible locations for the agents, and the edges $e\in E$ are the possible routes between locations.
- k agents labeled ${a}_{1},{a}_{2}\ldots {a}_{k}$, and every agent ${a}_{i}$ has a start vertex ${s}_{i}\in V$ and a goal vertex ${g}_{i}\in V$.

It is noteworthy that time is discretized into different points. At time point *t*_0_, agent $${a}_{i}$$ is in location $${s}_{i}$$. In this work, the classical Conflict-Based Search (CBS) algorithm was used for path finding^32^. On the low level, A* search is executed for all agents in a space-time graph with constraints marked as inaccessible vertices and returns single-agent paths consists of a sequence of locations $$\pi =({s}_{i},\ldots ,{g}_{i})$$. The high-level search explores a constraint tree (CT) that iteratively resolves conflicts by generating sets of constraints that restrict individual agents, each node $$N$$ contains a set of constraints imposed on the agents ($$N.{constraints})$$, a solution $$(N.{solution})$$, and the cost of solution ($$N.{cost})$$. The node $$N$$ is defined as a tuple of $$\left(\Pi ,{\rm{{\rm K}}},\Omega \right)$$, where:

- $\prod =\left({\pi }_{1}{,\pi }_{2},\ldots {\pi }_{k}\right)$ is a set of k paths, the path of the i-th microparticle from the starting position to the micro-chamber’s position is ${\pi }_{i}$;
- ${\rm{{\rm K}}}$ is the scalar cost value of $\pi (i.e.,{\rm{{\rm K}}}={\rm{{\rm K}}}\left(\pi \right)=\sum _{i\epsilon k}{\rm{{\rm K}}}({\pi }_{i}))$;
- $\Omega$ is a set of constraints. Constraints are represented by a tuple $({a}_{i},v,t)$ (or $({a}_{i},e,t)$)which states that agent ${a}_{i}$ cannot access vertex $v$ (or traversing edge $e$) at timestep $t$.

If conflicts persist after the low-level search, such as two or more agents occupying the same location simultaneously, the corresponding high-level node is labeled as a non-goal node, and the high-level search proceeds by introducing additional nodes that address the conflict through specific constraints. The two-level formulation enables CBS to explore fewer states than A* does, resulting in effective solutions with manageable computational complexity, which is suitable for this work.

### Supplementary Information

Descriptions and supplementary figures as well as descriptions of supplementary movies are provided in the Supplementary Information file.

## Supplementary information

Supplementary Information

Movie S1

Movie S2

Movie S3

Movie S4
