# Supplementary material for: Automated and collision-free navigation of multiple micro-objects in obstacle-dense microenvironments using optoelectronic tweezers
Source: Microsyst Nanoeng. 2025 Mar 17;11:49. doi: 10.1038/s41378-025-00892-9 (PMC11914063; doi:10.1038/s41378-025-00892-9)
Supplement: Supplementary file 1 — Supplementary Information [file 41378_2025_892_MOESM1_ESM.docx]

**Supplementary Information for**

Automated and collision-free navigation of multiple micro-objects in obstacle-dense microenvironments using optoelectronic tweezers

Lixiang Zheng,^1,†^ Gong Li,^2,†^ Henan Du,^2,3^ Zonghao Li,^2^ Bingrui Xu,^4^ Fan Yang,^4^ Yanan Mao,^4^ Jing Wei,^2^ Hainan Xie,^5^ Wei Xie,^5^ Rongxin Fu,^3,6,7^ Na Liu,^1,*^ Shuailong Zhang,^2,3,4,7,*^ Lianqing Liu,^8^ Wen Jung Li,^9^ and Yu Sun^10^

*^1^School of Mechatronics Engineering and Automation, Shanghai University, Shanghai, 200444, China*

*^2^School of Integrated Circuits and Electronics, Beijing Institute of Technology, Beijing, 100081, China*

*^3^Zhengzhou Research Institute, Beijing Institute of Technology, Henan, 450000, China*

*^4^Beijing Advanced Innovation Center for Intelligent Robots and Systems, School of Mechatronical Engineering, Beijing Institute of Technology, Beijing, 100081, China*

*^5^Optoseeker Biotechnology (Shenzhen) Co., Ltd., Shenzhen, 518055, China*

*^6^School of Medical Technology, Beijing Institute of Technology, Beijing, 100081, China*

*^7^Chongqing Institute of Microelectronics and Microsystems, Beijing Institute of Technology, Chongqing, 400000, China*

*^8^State Key Laboratory of Robotics, Shenyang Institute of Automation, Chinese Academy of Sciences, Shenyang 110016, China*

*^9^Department of Mechanical Engineering, City University of Hong Kong, Hong Kong, China*

*^10^Department of Mechanical and Industrial Engineering, University of Toronto, Toronto, ON, M5S 3G8, Canada*

*^†^These authors contributed equally.*

*Corresponding authors:

[liuna_sia@shu.edu.cn](mailto:liuna_sia@shu.edu.cn%20)  (N. Liu); [shuailong.zhang@bit.edu.cn](mailto:shuailong.zhang@bit.edu.cn) (S. Zhang)

**Supplementary Figures**

**
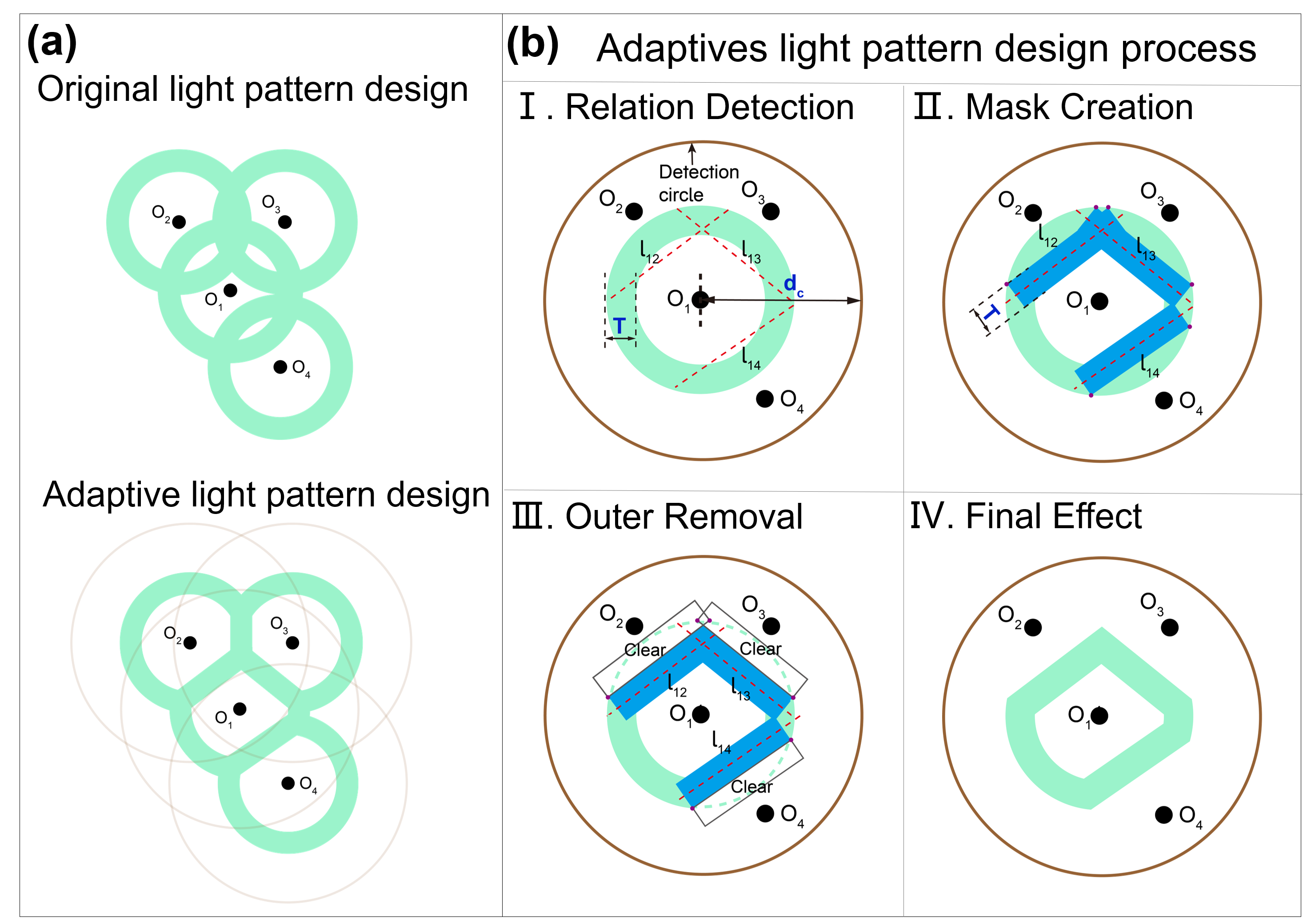
**

**Fig. S1.** **Adaptive light pattern strategy for multiple objects.** (a) Schematic diagram of the original light pattern design and the adaptive light pattern design. (b) Adaptive light pattern design process for multiple objects

**
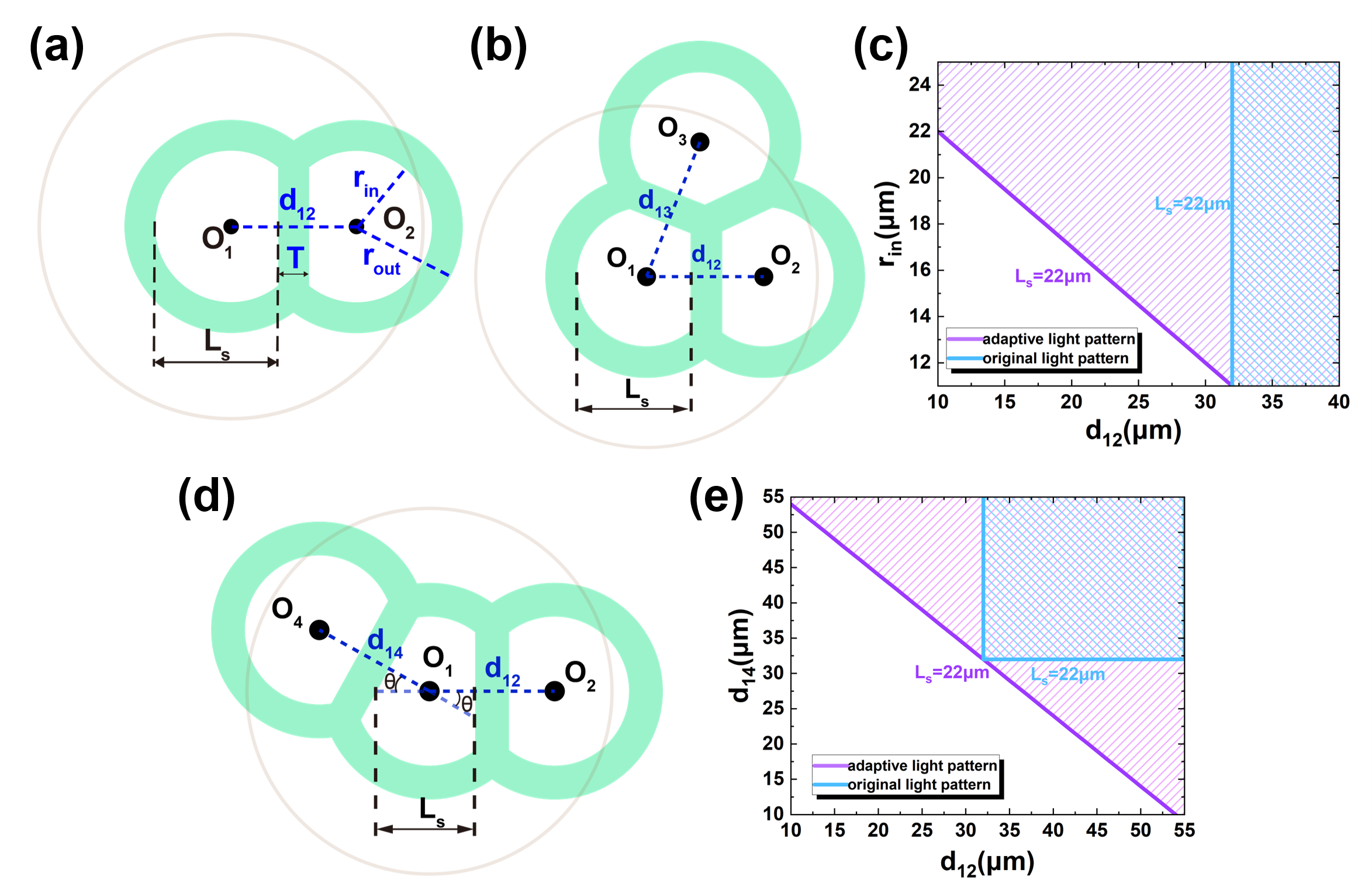
**

**Fig. S2. Adaptive light pattern design strategy reduces the need for safe distance required by algorithms.** (a) Schematic diagram of the adaptive light pattern design strategy for the two objects. (b) Schematic diagram of the adaptive light pattern design strategy for two-by-two encounters of multiple objects. (c) Variation of the *L_s_* of the light pattern *O_1_* in case (a) and (b) with the inner radius *r_in_* of the light pattern and the distance *d_12_* between the two light patterns under the adaptive light pattern design strategy (purple region) and the original design strategy (blue region). (d) Schematic of adaptive light pattern design strategy for multiple objects squeezing the same object. (e) (e)Variation of *L_s_* of the light pattern *O_1_* in case (d) with the inner radius *r_in_* of the light pattern and the distance *d_12_* between the two light patterns under the adaptive light pattern design strategy (purple region) as well as the original design strategy (blue region).


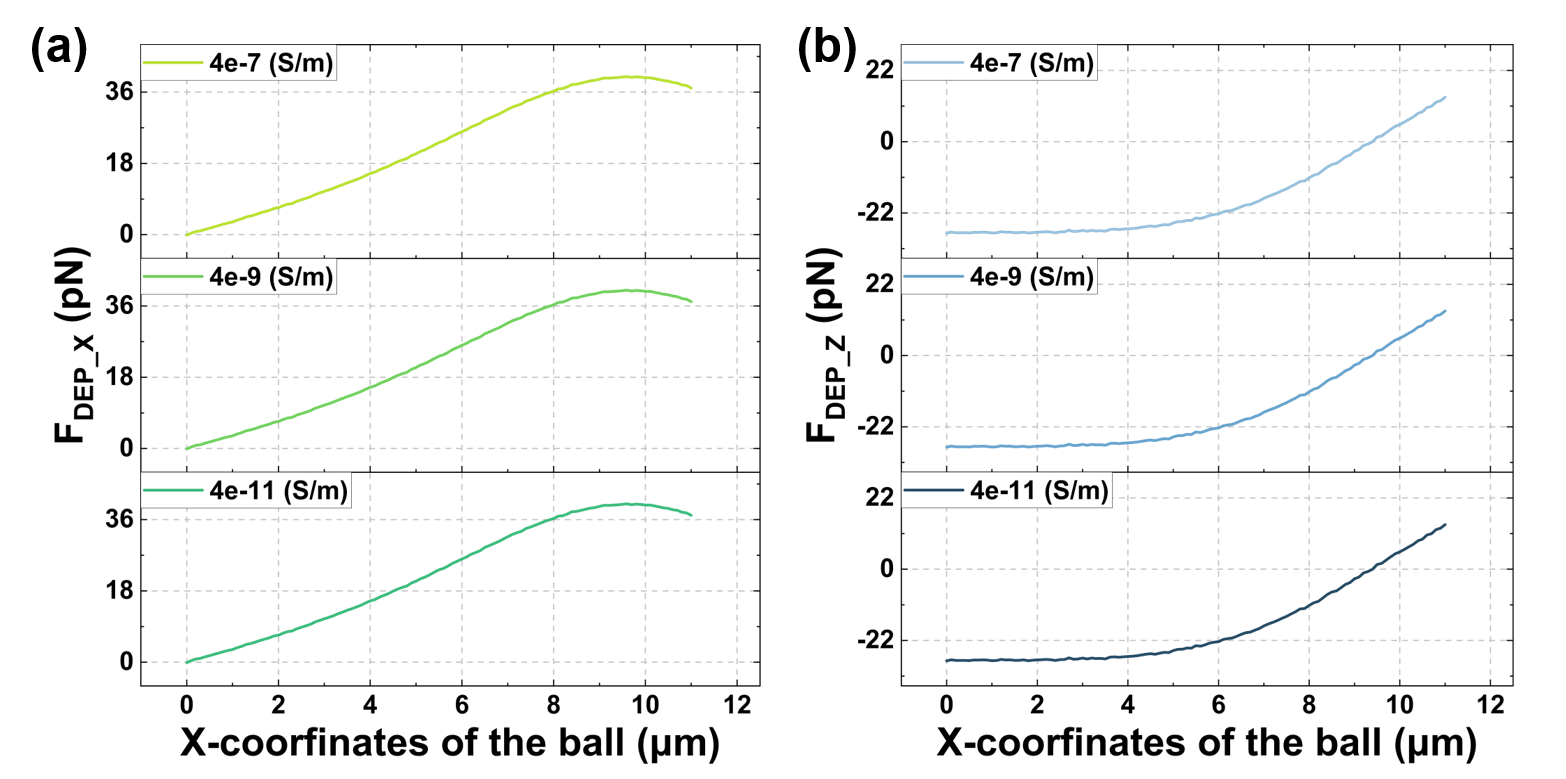


**Fig. S3. Simulation of microparticles with different conductivities (light pattern inner diameter: 22 µm).** (a) DEP force in the X direction exerted on microparticles at different positions. (b) DEP force in the Z direction exerted on microparticles at different positions.

**Supplementary Notes**

**Supplementary Note 1: Expansion of adaptive light pattern design strategies for multi-object**

The multi-object conflict problem can be decomposed into two types: multiple two-object conflicts and a series of two-by-two conflicts among objects. Therefore, an adaptive light pattern design strategy is essential to effectively address these scenarios.

Fig. S1a illustrates the scenario in which the light pattern of *O_1_* conflicts with three others, *O_2_*, *O_3_*, and *O_4_*, both with and without the adaptive light pattern design strategy. Figure S1b details the complete process of the adaptive design strategy for light pattern of *O_1_* as an example. This strategy establishes a detection circle around *O_1_*, with a radius defined by the critical conflict distance *d_c_* (where *d_c_ = r_in_ + r_out_*). Within this detection circle, other objects (*O_2_*, *O_3_*, *O_4_*) are considered to be in conflict with *O_1_*. In Fig. S1b I, *l_12_*, *l_13_*, and *l_14_* represent the centerlines between *O_1_* and the other objects, aiding in determining the relative positions of *O_1_* and the conflicting objects. In Fig. S1b II, the blue rectangular mask indicates the separation area for *O_1_* and the other objects. The width of the mask corresponds to the thickness *T* of the annular light pattern, while its length is determined by the intersection of the rectangle with the outer circle. This rectangular mask delineates regions based on the centerlines between *O_1_* and the other objects. In Fig. S1b III, the gray rectangular outline indicates the area where portions of the original light pattern for *O_1_*, located outside the blue rectangular mask, are removed to prevent interference with the other objects. Finally, Fig. S1b IV displays the adaptive light pattern corresponding to *O_1_*, wherein the original pattern is adaptively redesigned to maximize the controllable area inside, thus reducing the likelihood of operational failure.

The problem of multi-object in conflict two-by-two is relatively simple and can be expanded directly. Fig. S2a illustrates a fundamental scenario of a conflict between light patterns, where two light patterns, *O_1_* and *O_2_*, overlap. In this case, the parameter *L_s_* for *O_1_* is defined by the equation *L_s_ = r_in_ + (d_12_ - T)/2*, where *d_12_* is the distance between *O_1_* and *O_2_*. Fig. S2b presents t a situation in which a new conflicting object, *O_3_*, is introduced within the detection circle of *O_1_*, alongside the conflicting object *O_2_*. Since *O_2_* and *O_3_* are situated on the same side of *O_1_*, the parameter *L_s_* for *O_1_* is determined by the smaller of *d_12_* and *d_13_*, expressed as *L_s_ = r_in_ + (min (d_12_, d_13_) - T)/2*, where *d_13_* represents the distance between *O_1_* and *O_3_*. In both scenarios (a) and (b), the parameter *L_s_* for *O_1_* is defined by *r_in_* and a single distance *d*, categorizing them as Type 1 situations.

Fig. S2c illustrates the relationship between the parameter *L_s_* for light pattern *O_1_* under the adaptive light pattern design strategy (purple region) and the original design strategy (blue region), as a function of the inner radius *r_in_* and the distance between the two light patterns, *d_12_*. It is evident that within the purple region corresponding to the adaptive design strategy, both the radius *r_in_* and the distance *d_12_* can flexibly satisfy *L_s_* ≥ 22 μm. In contrast, under the original design strategy (blue region), *d_12_* must be at least 32 μm to ensure *L_s_* ≥ 22 μm.

In cases involving multiple two-object conflicts, the most challenging scenario occurs when several objects simultaneously conflict with a single central object, placing this object in a particularly critical position. Fig. S2d presents another scenario in which a new object, *O_4_*, is introduced within the detection circle of *O_1_* while *O_2_* is already present. Since *O_2_* and *O_4_* are positioned on opposite sides of *O_1_*, the parameter *L_s_* is determined by both *d_12_* and *d_14_*, where *d_14_* represents the distance between *O_1_* and *O_4_*. In this case, *L_s_* is given by the equation *L_s_ = (max (d_12_, d_14_) - T)/2 + (min (d_12_, d_14_) - T)/2 cos θ*. In the most extreme case, where *O_1_*, *O_2_*, and *O_4_* are collinear, the equation simplifies to *L_s_ = (d_12_ - T)/2 + (d_14_ - T)/2*. Since *L_s_* in this scenario is governed by two distances, *d_12_* and *d_14_*, it can be classified as a Type 2 situation. Fig. S2e illustrates the relationship between the parameter *L_s_* for light pattern *O_1_* in the Type 2 scenario under both the adaptive light pattern design strategy (purple region) and the original design strategy (blue region) as a function of distances *d_12_* and *d_14_*. In the purple region, both *d_12_* and *d_14_* satisfy *L_s_* ≥ 22 μm, whereas in the blue region, each distance must be at least 32 μm to ensure *L_s_* ≥ 22 μm. While the adaptive strategy may not ensure sufficient manipulation space under extreme conflict conditions, it significantly improves upon the original design.

**Supplementary Note 2: Effect of Conductivity Magnitude Variation of Polystyrene Microparticles on Numerical Simulations**

In this study, the impact of the conductivity of polystyrene microparticles on numerical simulation results was evaluated. According to the experimental setup, the conductivity of the microparticles must be lower than that of the solution to experience negative DEP forces under an external electric field. To assess the potential effects of variations in conductivity, simulations were conducted for microparticle conductivities of 4 × 10^-7^, 4 × 10^-9^, and 4 × 10^-11^ S/m.

The simulation results indicated that the forces acting on the microparticles in both the X and Z directions remained nearly unchanged within this range of conductivity values (see Fig. S3). These findings suggest that, under the experimental conditions (i.e., the microparticle conductivity is lower than that of the solution, and the microparticle experiences negative DEP forces), changes in the conductivity magnitude have negligible effects on the numerical simulation outcomes. Therefore, we conclude that slight variations in conductivity do not significantly impact the results of this paper, and this factor has been adequately considered in this work.

**Supplementary Tables**

**Table S1.** Experimental data of particle trap by light patterns of different size.

| Inner diameters (Φ) | Number of successes | Total number |
| --- | --- | --- |
| 14μm | 0 | 30 |
| 16μm | 4 | 30 |
| 18μm | 5 | 30 |
| 20μm | 14 | 30 |
| 22μm | 30 | 30 |
| 24μm | 30 | 30 |

**Table S2.** Simulation parameters of the OET device.

| Parameters | Descriptions | Values |
| --- | --- | --- |
| σ_m_ | Conductivity of liquid medium | 5.5×10^-6^ S/m |
| σ_Si-illuminated_ | Conductivity of illuminated a-Si:H | 1×10^-4^ S/m |
| σ_Si-dark_ | Conductivity of dark a-Si:H | 1×10^-6^ S/m |
| σ_PS-microparticle_ | Conductivity of PS microparticle | 4×10^-11^ S/m |
| ε_m_ | Permittivity of liquid medium | 80 |
| ε_Si-illuminated_ | Permittivity of illuminated a-Si:H | 11.7 |
| ε_Si-dark_ | Permittivity of dark a-Si:H | 11.7 |
| ε_PS-microparticle_ | Permittivity of PS microparticle | 2.55 |

**Supplementary Movies**

**Movie S1:** Experimental video showing the failure of microparticle's manipulation due to cross-interference between two light patterns (Fig.2a).

**Movie S2****:** Experimental video showing the translational and rotational motion of two microparticles manipulated by adaptive light patterns (Fig.4f, Fig.4g).

**Movie S3:** Experimental video showing the automatic transportation of 10 small microparticles to pass through microchannels in between obstacles and reach designated microchambers (Fig.6a).

**Movie S4:** Experimental video showing the automatic transportation of 5 small microparticles through custom selection of goal positions.
